# Assessment of a two-year school-based physical activity intervention among 7-9-year-old children

**DOI:** 10.1186/1479-5868-8-138

**Published:** 2011-12-20

**Authors:** Kristjan Thor Magnusson, Ingvar Sigurgeirsson, Thorarinn Sveinsson, Erlingur Johannsson

**Affiliations:** 1 Research Centre for Sport and Health Sciences, School of Education, University of Iceland, Reykjavik, Iceland; 2 Faculty of Teacher Education, School of Education, University of Iceland, Reykjavik, Iceland; 3 Research Centre for Movement Sciences, School of Health Sciences, University of Iceland, Reykjavik, Iceland

## Abstract

**Background:**

Physical activity (PA) in children has declined in recent decades, highlighting the need for effective intervention programs for school-aged children. The main objective of this study was to assess to what extent PA during and after school hours changed among children who received a progressive two-year long intervention *vs. *that of children who only received general curriculum-based PA.

**Methods:**

A cluster randomized intervention study was conducted and six elementary schools randomly assigned to serve as control- or intervention schools. All children attending second grade (mean age = 7.4 years - born in 1999) were invited to participate in the fall of 2006 (N = 320, 82% participated), again in 2007 (midpoint) and 2008 (end of intervention). The intervention consisted of multi-component PA-intervention during school hours and was conducted by teachers at each intervention school. PA was assessed by means of accelerometers and subjectively at the intervention schools via teachers' PA log-books.

**Results:**

There was no difference in PA intensity (minutes of moderate-to-vigorous physical activity - min of MVPA) between the two study groups at baseline, but children in the intervention schools were more physically active at moderate-to-vigorous intensity compared to those in control schools after one year of intervention (mean difference of MVPA_log-minutes_: 0.61, 95%CI: 0.02, 1.20, p = 0.04). Moreover, the model for minutes of MVPA during school hours, showed a significant three-way interaction between time at mid-point, group and gender (mean difference of MVPA_log-minutes_: 1.06, 95%CI: 0.15, 1.97, p = .02), indicating a significantly greater increase among the boys in the intervention schools compared to girls. No difference in PA was detected between the study groups at the end of the study period after two years of intervention.

**Conclusions:**

The results suggest that the objective of increasing PA at school was met after one year of intervention, and it was more pronounced among boys. The lack of increase at the end of the study period suggested that any increase in PA during school may highly depend on both motivation and training of general teachers. Boys may respond better to PA interventions such as the one described in this study.

## Background

A vast amount of research has confirmed that physical inactivity is an important factor in the causal mechanisms of major chronic diseases such as obesity, cardiovascular diseases, diabetes, and more [1]. In light of these negative health effects of an inactive lifestyle it is alarming to find information which indicates a decline in children's physical activity in recent decades [2]. Secular trends of physical activity patterns in Icelandic children are scarce, but results from a cohort of 9 and 15-year old children showed that only a small proportion of students at this age reached the recommended physical activity guidelines [3]. Consequently, this highlights the need for effective intervention programs that focus on increasing both the amount and intensity of physical activity related behavior among school-aged children. This need is even more evident in light of the progression and prevalence of overweight and obesity among Icelandic children in recent years [4].

Schools are generally considered ideal settings for the promotion of physical activity and healthy lifestyle for several reasons, such as the ease of repeated access to a large number of children, the somewhat controlled environment of the school, and the general lack of cost to families [5,6]. However, recent systematic reviews of school-based physical activity intervention studies show somewhat disappointing results [7,8]. These reviews highlight the need for studies of high methodological quality. Common weaknesses in school-based interventions studies involve a lack of objective measures of physical activity, the quality of physical activity administered and lack of statistical power to detect differences. The majority of school-based physical activity interventions carried out to date have relied on self-reported physical activity. This may have caused differential misclassification in levels of physical activity introduced by social desirability bias, which can be induced by the intervention itself [9]. However, there are intervention studies that measured children's and adolescents' physical activity with objective instruments such as accelerometers [10-15] but they show inconsistent results. A recent study reported a highly effect intervention-program among elementary school children which relied on a multi-component physical activity program, including re-structuring three physical education lessons each week and adding two extra lessons a week, daily short activity breaks, and physical activity homework [15]. Despite being a very promising study it may prove difficult for an intervention of this sort to become widespread because of the cost to schools which must allocate more of their resources and time to provide students with two extra PE lessons per week.

Due to the potential influence of local environmental factors, culture and educational setting on any school-based intervention program, it is important to build an evidence base within each country. In Iceland, no study of this kind has been conducted to date. It is thus important to provide knowledge to this field that is applicable to local circumstances.

The research question asked in this study was how much a two-year progressive teacher-led physical activity intervention could affect physical activity during school hours and physical activity after school hours among 7-year Icelandic children. Thus, the objectives of this study were to compare changes in volume and intensity of physical activity among the group of intervention children to physical activity levels of children who only received general curriculum-based physical activity (controls) and further, to assess whether the intervention effect on physical activity was modified by gender or BMI?

## Methods

### Study Design and Participants

**Figure 1:**
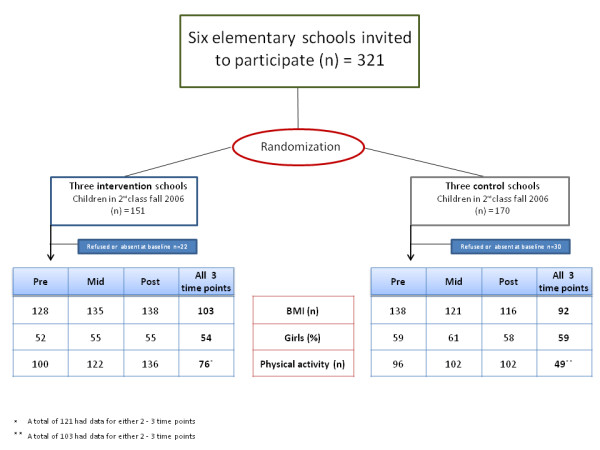
**Chart describing school and participant flow through the study**.

Eight months before the baseline measurements three pairs of schools in the city of Reykjavik were selected and matched on size, i.e. number of students and total number of grades. Thus, four large schools with grades one to ten and two schools with only grades one to seven, with at least 30 students entering the second grade in 2006 were sent letters of participation, which they all accepted. Then, one of the schools in each pair was randomly selected to serve as an intervention school, leaving the other as a control school. All children attending second grade (N = 321, born in 1999) were invited to participate and to hand in a written consent form. Figure 1 shows the flow of participants through the three measurement sessions of the study.

The intervention started immediately after baseline measurements. All children in the second grade of the participating intervention schools received the intervention, regardless of whether they consented to undergo physical measurements or not. The intervention focused on two major components of a healthy lifestyle: physical activity and a healthy diet for all. The latter component is not described here. The implementation of this study was approved by The National Bioethics Committee and the Icelandic Data Protection Commission (VSN b200605002&03).

The same three PE teachers (all certified PE teachers) at each intervention school took part in the study throughout the two-year intervention period. At any given time a total of eight general teachers implemented the intervention, three in two of the intervention schools, but two in the third school.

### Background to intervention

The foundation of the school-based physical activity intervention in this study was built upon the constructs of social cognitive theory [16]. According to the theory we may learn new behavior via observational learning of various social factors and interactions in our environment. We are likelier to imitate and adopt certain behaviors if we observe positive, desired outcomes in the behavior under examination. We are even likelier to pursue these behaviors if they are modeled by someone we know or someone we relate to. Individuals who repeatedly play the role of such models are teachers, who often possess important qualities, which they utilize to positively affect their students' learning. This process has been proven useful for understanding the nature of physical activity by considering a person's experiences, behavior skills and the context in which the person is expected to be active [17].

### Intervention objectives and implementation

The primary objective of the physical activity intervention was to progressively increase the amount of physical activity behavior at school such that all children in the intervention schools would have the opportunity to engage in some form of physical activity for a minimum of 60 minutes during school hours no later than one year after baseline measurements. The students enrolled were to have opportunities to engage in physical activity during PE lessons, recess and also during classes where physical activity was to be integrated into various subjects on the general curriculum. The primary objective of the intervention is in line with the current Icelandic physical activity guidelines, which recommend that children partake in Moderate-to-Vigorous Physical Activity (MVPA) for no less than 60 minutes every day of the week [18]. The guidelines are built upon evidence that suggests that at least one hour of MVPA per day provides desirable health benefits for children and youth [19].

Our implementation approach was aimed at both encouraging teachers to perform physical activity with their students and to build their necessary skills to become the implementers of change needed to positively affect the children's lifestyles. This approach was intended to serve as the bridge between theory (social cognitive theory) and intervention effects. The researchers provided a platform for a reflective dialogue and discussion of how these goals could be reached by means of collaborative effort and support. The foundation of the collaborative approach harmonizes with the strategy labeled as professional learning community (PLC). In brief, PLC is a learning platform promoting collaborative learning amongst colleagues within the same work environment. It is commonly defined as the community in a school where teachers and administrators continuously seek and share learning and subsequently act on what they learn [20]. A number of attributes are present in such a community and the researchers' goals were to have the participating teachers and principals all share the same vision and values whilst working towards meeting the intervention objectives.

Bimonthly, throughout the intervention period, the investigators organized a workshop-meeting where all the involved teachers from each intervention school met for about 2-3 hours. These meetings had three main goals. Firstly, to provide an opportunity for the team of teachers in all three intervention schools to meet and engage in dialogue with their colleagues about the evolvement of the intervention. Secondly, these meetings were intended to provide teachers with information and expert consultations on the benefits of both physical activity and healthy lifestyle via informal lectures on relevant topics. Thirdly, these meetings were to serve as platforms where teachers and researchers would collectively work together to overcome various barriers related to but not limited to the implementation and integration of physical activity into daily life at each school.

The teachers at the intervention schools were provided access to physical activity equipment intended to be used during regular school lessons. This included a cart with different sized foam, plastic and rubber balls, different colored vests, and cones. Teaching materials promoting physical activity, such as books and DVDs on classroom workouts and cooperative activity games et cetera were also provided. One day of on-site counseling led by a school-based PE expert was conducted at each school in the spring of 2007, where teachers learned innovative approaches to integrating physical activity into the daily curriculum. Thus, the teachers received input and ideas as to how they could better utilize available equipment and the study material in their respective environments. After the first year of intervention an additional PE lesson was introduced at the intervention schools. The PE teachers at each of the intervention schools carried out this additional lesson, which was specifically tailored to suit all children while maintaining a high level of intensity. All six schools that participated in the study followed the general physical activity curriculum, compulsory on the national level, consisting of two 40-minute PE sessions per week, in addition to two swimming lessons per week taught over the course of a six-week period any time during the school year.

Once each semester the investigators formally teamed up with the principals of the three intervention schools to discuss the progress of the intervention. During these meetings the researchers updated the principals on what had taken place during the previous teacher meetings and encouraged a continuous on-site dialogue between the principals and the teachers who implemented the intervention. In between the meetings described above the researchers, teachers, and principals of the intervention schools communicated via e-mail or phone.

### Physical activity logbook

During the intervention period the teachers at each intervention school kept a log of the estimated amount of supervised physical activity related behavior they carried out with their students as a group (an assessment of the intervention implementation). Thus, they estimated the number of minutes they engaged in any type of physical activity during school hours, including PE lessons, swimming lessons, outdoor play/teaching other than recess time, and active transport during school such as on field trips. The mean minutes of supervised physical activity/day at school were calculated for each school.

### Group interviews with teachers

To identify important catalysts of and barriers to the intervention the researchers conducted semi-structured group interviews in all three intervention schools in the spring of 2008. All teachers (N = 11) in each school (n = 4 - 4 - 3) participated and provided input. The teachers reflected on lessons learned throughout the process, and on anything that affected the implementation of the intervention at the schools. The teachers were also asked to comment on any changes in factors related to class discipline, morale, and unity over the course of the study.

### Primary outcome measures

Accelerometers were used (Actigraph™ GT1M monitors) to assess both volume and intensity of physical activity during waking hours for seven consecutive days, five weekdays and two weekend days. The sampling epoch time was 60 s. Measurements were performed between mid-September and late November pre-intervention in the fall of 2006, in the fall of 2007 and 2008. Measures were calculated for children that met the inclusion criteria of having worn the accelerometer for at least 85% of the approximately 6-hour long school day and a total of 10 hours per day, for a minimum of two weekdays. This inclusion criteria allowed some flexibility in situations such as if children were attending swimming lessons during school hours or failed to attach it back on after such a lesson. Several cut-point thresholds for estimating MVPA in children by means of accelerometers have been proposed [21-25], yet there is no consensus on where to place these MVPA cut-point values. We defined MVPA as all activity above 2000 cpm as has been reported previously [26,27]. This cut-point is comparable with the one proposed by Evenson et. al [25], which was recently argued to have the best sensitivity/specificity ratio compared to several other common cut-offs [28].

### Covariates

Gender was recorded and height and weight were measured to the nearest 1 mm and 0.1 kg with participants wearing light clothing (t-shirt and underwear). Body mass index (BMI, kg/m^2^) was calculated and the actual number used as a covariate in the multilevel models.

### Data Analysis

We built multilevel regression models using R version 2.11.1 (http://www.r-project.org/) to assess changes in objectively measured physical activity volume (cpm) and intensity (MVPA) over time. This method of data analysis is appropriate wherever data is clustered within groups, violating the independence of observations assumption [29]. Repeated measures are one example of such data structures and in our case the data were also clustered within schools, implying that data points may be correlated both within students as well as within schools.

Two types of multilevel-regression models were developed. Firstly, a basic model to assess the intervention effects on physical activity with participants and schools treated as random factors and group and time as independent variables. Secondly, an adjusted model built upon the former model but with the addition of gender and BMI as covariates (two and three way effect-measure modification was also assessed for time, gender and BMI but only interactions yielding statistically significant results were reported in the final models. Thus, these models estimated the variance of the outcome measures by taking into account and allowing intercepts to vary within students, random intercepts across students, and it allowed random intercepts for the six different schools that participated in the study. The unconditional (no independent variables - only random factors entered into the model) three-level models were run to estimate the total variance at all three levels. The intraclass correlations can be defined according to the proportion of variance that occurs at each level. The change in variance from the unconditional model to the best conditional (adjusted) model at each level can thus be presented as the percentage of variance explained at each particular level.

A priori, the study was powered to detect a medium effects size (0.25 SD units) of any outcome measure with 80% probability, using ANCOVA to compare the post intervention measurements adjusting for baseline, not taking the clustering within schools into account. The sample size estimated was 175 children at the end of the study period. We assumed a participation rate of 75% and due to the length of the study we assumed an attrition rate of about 20%. Thus all 321 children entering second grade in 2006 from six schools were offered the chance to participate in order to provide sufficient power to test the null hypothesis.

We ran an intention to treat analysis and all participants were included regardless of how much time they accumulated in their respective class during the intervention period between the fall of 2006 and fall of 2008. The multilevel analysis included subjects with missing data points but imputation was not performed.

## Results

A total of 196 children produced usable physical activity data after the first session in 2006 while 52 children (16%) did not consent and 73 children (27%) failed to meet the accelerometer criteria set forth prior to the study (described above). Similarly, 224 children yielded usable data after the session in 2007, 78 new participants entered the study at that time point, but 50 children (25%) were lost to follow up from the year before, either due to failing to meet accelerometer criteria or they did not participate in the measurements. At the end of the study period 239 children produced usable data, 35 of whom had either not produced usable data in the year before and entered the study again, or entered for the first time. However, 20 children (9%) from the previous year were lost due to their failure to meet the accelerometer criteria or due to not participating in the measurements.

### Baseline status

Table 1 shows the characteristics of the study population at baseline. There was a statistically significant difference in BMI between the two study groups at baseline, where children of the control group had higher mean BMI value (95%CI; 0.08, 1.16) (Table 1.). Data on socioeconomic status (SES) estimated via questionnaire in the fall of 2006 and fall of 2008 suggested no difference between the study groups in median income of the parents, but only about half of the study population answered the question.

**Table 1:** Descriptive statistics for the study sample at baseline

	Baseline - 2006					
	
Variables	Intervention group			Control group		
	
	Total (n = 100)	Boys (n = 48)	Girls (n = 52)	Total (n = 96)	Boys (n = 39)	Girls (n = 57)
	
*Age and body composition*	*median(QD)*	*median(QD)*	*median(QD)*	*median(QD)*	*median(QD)*	*median(QD)*
Age (years)	7.3 (0.2)	7.3 (0.3)	7.3 (0.3)	7.4 (0.2)	7.3 (0.2)	7.4 (0.2)
Height (cm)^a^	127.6 (5.1)	127.9 (4.9)	127.3 (5.3)	127.2 (5.2)	128.4 (5.1)	126.4 (5.1)
Weight (kg)	25.9 (2.4)	26.2 (2.0)	25.4 (2.7)	26.8 (2.4)	27.5 (2.2)	25.7 (2.4)
BMI	15.8 (1.1)	15.8 (0.9)	15.8 (1.2)	16.3 (1.0)	16.6 (0.7)	16.0 (1.1)

***Physical activity - accelerometers***	*median(QD)*	*median(QD)*	*median(QD)*	*median(QD)*	*median(QD)*	*median(QD)*

cpm at school^b^	721.6 (210.1)	790.8 (201.4)	656.5 (198.6)	655.1 (220.7)	728.8 (177.0)	606.0 (234.4)
MVPA at school (min)^c^	36.3 (14.3)	45.2 (16.1)	30.2 (12.6)	32.2 (11.6)	40.7 (9,5)	26.8 (15.3)
MVPA after school (min)^c^	29.7 (11.2)	29.7 (12.5)	29.8 (10.5)	31.6 (15.0)	40.8 (12.0)	29.1 (17.3)

^a^mean and standard deviation, not median (QD)

^b^cpm:counts per minute

^c^MVPA: moderate-to-vigorous physical activity defined as >2000 accelerometer counts per minute

### Physical activity - accelerometers

**Figure 2:**
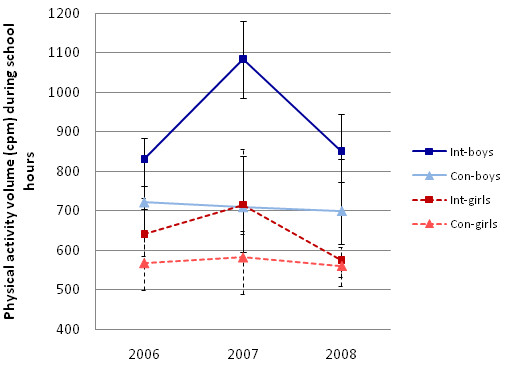
**Median cpm during school hours at baseline 2006, fall 2007, and post intervention in 2008 with estimates of the 95% CI around the median**.

**Figure 3:**
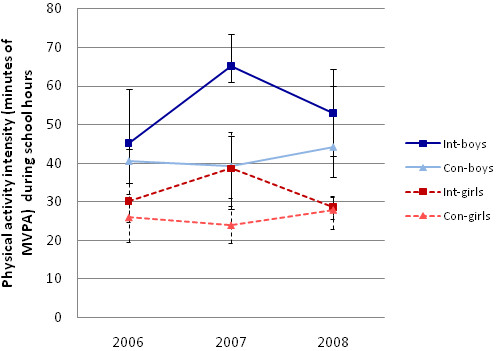
Median minutes of MVPA during after-school hours at baseline in 2006, fall 2007, and post intervention in 2008 with estimates of the 95% CI around the median

Two basic models showing the estimated unadjusted effects of the intervention on physical activity cpm during school and minutes of MVPA during school hours are shown in Table 2. Children in the intervention schools were significantly more active after one year of intervention (at the mid-point of the intervention period) compared to children in the control schools (p > .0001), but there was no significant difference in either volume (p = .10) or intensity (p = .71) of physical activity at the end of the two-year study period. Analogous models, but adjusted for the effects of gender and BMI, are shown in Table 3. These models are complemented by the trajectories depicted in Figure 2 and Figure 3, which show the true median values in the non-transformed units. The results for the adjusted models were analogous to those for the basic models and showed significant difference in volume of physical activity (cpm) during school hours (p > .0001) after one year of intervention, but no difference at the end of the two-year intervention period (p = .61). In addition the adjusted model showed that boys were more active compared to girls (p > .0001) and children with higher BMI were less active than children with lower BMI (p = .01), independent of study group and time. The adjusted model for minutes of MVPA during school hours showed analogous results, but with the addition of a significant three-way interaction between time at mid-point, group and gender (p = .02). Thus, the intervention children accrued significantly more minutes of MVPA during school hours after one year of intervention (p > .0001) but the increase was significantly greater in boys compared to girls (p = .02), by about 10 minutes (Figure 2). The adjusted multilevel model for square-root-transformed cpm explained 20.6% of the variance existing at the school level, 24.6% of the variance at the child level, but less of the within-child variance, or 10.5%. Similarly, the adjusted model for the square-root-transformed MVPA during school hours explained 14.1% of the total variance at the school level, 29% of the variance at the child level, and mere 6.6% of the within-child variance.

**Table 2:** Physical activity cpm during school hours (square root transformed) is shown as a function of time, group status, BMI and sex. Repeated measures mixed effects models were built to contrast the two study groups, intervention vs. control, over time with regard to physical activity during school time, controlling for the clustering of data structure.

	Basic models*					
	counts per minute(cpmschool)			MVPAschool (mins/day)		
	β	CI	p-value	β	CI	p-value
Constant	25.25	(22.96, 27.54)	>.0001	5.47	(4.57, 6.37)	>.0001
Group^a^	1.13	(-3.43, 5.70)	.53	0.62	(-1.18, 2.42)	.39
Time(mid)^b^	-0.31	(-1.26, 0.64)	.52	-0.06	(-0.40, 0.27)	.72
Time(post)^b^	-0.60	(-1.56, 0.36)	.22	0.32	(-0.03, 0.67)	.07
Time(mid) x Group	3.60	(2.31, 4.89)	>.0001	0.98	(0.53, 1.43)	>.0001
Time(post) x Group	0.24	(-1.04, 1.53)	.71	-0.39	(-0.85, 0.07)	0.1

The constant is the mean square-root cpm during school for the control schools (group = 0), at baseline (time = 0).

^a ^reference: controls

^b ^reference: time at baseline

^c ^reference: girls

**Table 3:** Minutes of MVPA during school hours (square root transformed) is shown as a function of group status, time, BMI and sex. Repeated measures mixed effects unconditional vs. conditional models controlling for the clustering of data structure contrast the two study groups over time with regard to time spent in MVPA during school hours.

	Adjusted models*					
	counts per minute(cpmschool)			MVPAschool (mins/day)		
	β	CI	p-value	β	CI	p-value
Constant	26.77	(23.67, 29.85)	>.0001	5.79	(4.58, 6.99)	>.0001
Gender	3.71	(3.06, 4.35)	>.0001	1.75	(1.22, 2.28)	>.0001
Group	0.76	(-3.31, 4.84)	.63	0.79	(-0.93, 2.51)	.27
BMI	-0.18	(-0.32, -0.04)	.01	-0.06	(-0.11, -0.01)	.02
Time(mid)	-0.21	(-1.15, 0.73)	.66	0.07	(-0.34, 0.49)	.72
Time(post)	-0.36	(-1.32, 0.59)	*.46*	0.42	(-0.02, 0.86)	.06
Time(mid) x Group	3.63	(2.35, 4.91)	>.0001	0.61	(0.02, 1.20)	.04
Time(post) x Group	0.33	(-0.94, 1.60)	.61	-0.48	(-1.26, 0.05)	.07
Gender x Group				-0.74	(-1.47, -0.01)	.05
Time(mid) x Gender				-0.36	(-1.04, 0.32)	.30
Time(post) x Gender				-0.11	(-0.83, 0.61)	.76
Time(mid) x Group x Gender				1.06	(0.15, 1.97)	.02
Time(post) x Group x Gender				0.72	(-0.21, 1.66)	.12

The constant represents the mean value for the control group, but only for girls (gender = 0) and those with an average value of BMI

^a ^reference: girls

^b ^reference: controls

^c ^reference: time at baseline

**Figure 4:**
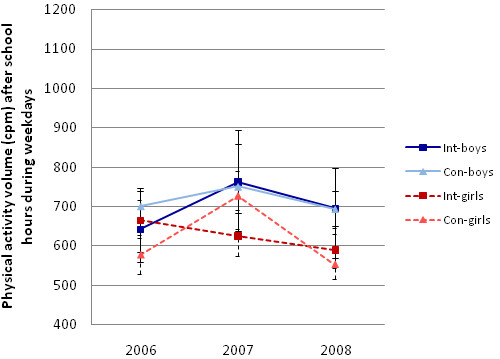
**Median cpm during school hours at baseline 2006, fall 2007, and post intervention in 2008 with estimates of the 95% CI around the median**.

**Figure 5:**
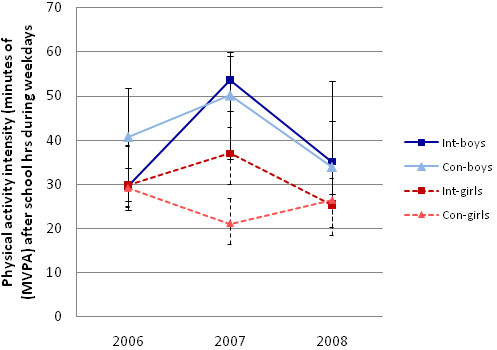
**Median minutes of MVPA during school hours at baseline in 2006, fall 2007, and post intervention in 2008 with estimates of the 95% CI around the median**.

The same types of models (basic and adjusted) were run to observe possible change in cpm and minute of MVPA during after-school hours on weekdays over time. The unadjusted trajectories are depicted in Figure 4 and Figure 5. The adjusted model for cpm during after-school hours yielded no significant difference between intervention and control schools at baseline (p = .33), at mid-point (p = .09) or at the end of the intervention period (.91). However, boys were more active than girls (p = .001) during after-school hours and those with higher BMI were less active (p = .02), independent of study group and time. Similarly, the adjusted model for the square-root-transformed minutes of MVPA during after-school hours showed no significant difference between the two study groups at baseline (p = .58), at mid-point (p = .59) or at the end of the study period (p = .50). Boys accrued on average more minutes of MVPA during after-school hours compared to girls (p > .0001), but there was no difference in minutes of MVPA during after-school hours by value of BMI (p = .40), independent of study group and time. It should be noted that conducting the analyses above on the original cohort only, excluding children who had missing values of data at any of the three time points, yielded comparable results, but with power too low to detect some of the significant relationships detected by the models described above.

### Physical activity during school hours - subjective assessment of implementation

**Figure 6:**
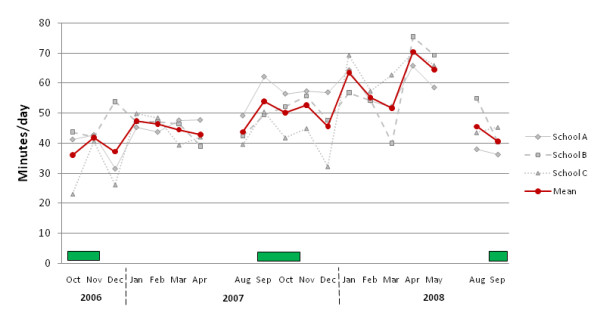
**Subjective estimation from teachers' physical activity log book of time spent doing physical activity (under teacher's supervision) at school following baseline measurements in 2006 until end of intervention period**. Each of the intervention schools started their respective intervention following the baseline-measurements. Data is not shown for September 2006, May 2007 and October 2009 because only one or none of the intervention schools registered physical activity during those months. The dark squares represent the time during which physical activity was being assessed with accelerometers.

The estimated mean minutes of supervised/integrated physical activity conducted by teachers in each intervention school per day are depicted in Figure 6 (overall mean, and mean for each school). These results suggested an upward trend in physical activity implementation conducted during school hours over the course of the study but with a drop in physical activity implementation at the end of the study.

Findings listed as key themes that were identified during teacher interviews in the spring of 2008, along with sample quotes from the teachers involved in intervention implementation, are shown in Table 4. Both groups of teachers were generally positive when evaluating their experiences during the time they implemented the intervention. Overall they enjoyed the progressive implementation approach of the intervention allowing time for on-site trial and error.

**Table 4:** Results showing common themes that came up during three semi-structured interviews where benefits, facilitators and barriers of the intervention implementation were discussed

Teachers interviewed (N = 11)	Common themes	Sample quotes
General teachers (n = 8)	Benefits of implementation • Positive attitudes towards PA • Increased PA during school hrs • Changed attitude towards PA among teachers • Calmness in class after intervention activities • Positive effect on student productivity in other subjects • More unity in class Facilitators • Satisfaction with training meetings • Satisfaction with on-site counseling • Extra PE lesson Barriers to implementation • Steep learning curve in the beginning • Competing curriculum demand and specific subject requirements • The Icelandic winter weather • Teachers must be open to changing their own habits	"I think that outdoor play is much more natural to them now than it was when we started the intervention" "The training meetings were very informative and it was also important to me to feel like I was part of a team" "The children are more willing to do work in class after we have gone outside to play, they seem to be more receptive" "As a new teacher I can say that this way of integrating PA into the daily routine at school is something I will continue doing, everyone benefits from this, the students and I alike. "In the beginning it was very difficult for me to start thinking outside the box in order to increase the amount of PA. The objectives looked almost impossible to achieve in the beginning" "Sometimes the children came to school not properly dressed. They were not ready for outdoor activities in rain or snow"

PE teachers (n = 3)	Benefits of implementation • Readiness of all students to partake in PA • Willingness to explore new sports Facilitators • Extra PE lesson • Positive attitude of school principal • Good collaboration with participating teachers • Positive attitude of teachers Barriers to implementation • Tightly booked gymnasium facilities and quality of the school property (one intervention school only)	"There were never any problems with this class. No one forgot to bring their sports clothing, everyone was always ready to play" "The extra PE lesson during the latter year was very successful" "For me it was very enjoyable to collaborate with other teachers. I think the general teachers also enjoyed seeing their students in a different environment, such as in the gymnasium"

## Discussion

Results from accelerometers showed children in the intervention schools being more active compared to those in control schools after one year of intervention. The difference at that time point was driven by boys in the intervention group, who at that point had increased their amount of MVPA significantly more than the intervention girls. No group difference was detected at the end of the intervention period a year later. Boys in both groups were consistently more active than girls at all three time points, and those children with higher BMI were less active during school hours and after school hours, but there was no association between BMI and physical activity during after-school hours.

It is important to evaluate the intervention process of every intervention study conducted before the efficacy of the program on other biological or social variables is assessed. Was the intervention conducted as planned, and if so, to what extent were the objectives met? To our knowledge no study has used the combination of methods described herein to assess changes in school-related physical activity during a two-year school-based intervention program. It strengthens the results that there seems to be harmony between both objective assessment of physical activity and subjective assessment of physical activity implementation, to the extent that they can be compared. Both measurements show a parabolic curve-shape when the three time points where physical activity was objectively assessed are contrasted. Perhaps these findings are positive because objective and subjective measurements have often showed inconsistent, even contradictory, results, both in observational and experimental studies [11,30].

The purpose of the extra PE lesson in the second year of the intervention was to considerably increase the amount of time where all participating children would get an opportunity to partake in physical activity of greater intensity. However, it seems clear that the increase in number of minutes engaged in MVPA during school hours in the fall of 2007 is driven by the boys being considerably more active at moderate-to-vigorous intensity, by on average about 10 minutes during school hours. These results are somewhat in line with results from the M-SPAN study, which conducted a two-year intervention focused on an environmental and policy-driven approach in middle schools in San Diego County, California where they did not see positive intervention effects on physical activity in girls [31]. The authors claimed that challenges were anticipated since girls are generally less active than boys but the reasons for these differential effects were nevertheless unclear. Otherwise, there is little evidence for boys and girls responding differently to school-based interventions as well as to different components of the interventions [7]. Our results nonetheless do suggest a future effort be made to test various gender-specific strategies given the gender-specific differences in the determinants of physical activity [32-34] and the results presented here. These results are nonetheless disappointing because the teachers reported during interviews that they had emphasized activities that they intended to be equally suitable for both genders.

Studies have previously reported conclusive evidence for gender specific differences concerning the amount of physical activity performed by children and adolescents [35-37], which is also confirmed at all time points in this study. In light of this fact we emphasized the importance that activities performed during PE and during regular class hours would suit boys and girls equally. None of the classroom teachers (all female) reported they had experienced gender differences in participation in the numerous activities performed during the intervention phase when asked specifically about it during the group interview sessions in the spring of 2008. However, there are several plausible explanations for this seemingly different response between the genders. First, the teachers' assessment may be wrong! Secondly, perhaps girls were not as active as boys during recess periods, when the children were not under surveillance by their teachers, in the fall of 2007. Another explanation may partly lie in how differently boys and girls may perceive barriers and facilitators of physical activity. A recent study of 350 adolescents in Maryland in the US, showed that when it came to performing physical activity, adolescent girls were more sensitive to their environment and perceived more barriers than boys [38]. If this holds true for younger children then perceived environmental barriers may have contributed to this differential rate of increase in MVPA during school hours in fall of 2007. It is also worth mentioning that results from other intervention studies that have used objective measures to assess MVPA have shown significant increase in MVPA after and during the intervention, but do not show differential intervention effects on intensity nor volume between boys and girls [14,15].

There are several possible explanations for the drop in school-related physical activity recorded by the teachers at the end of the study. We believe that the primary reason for this is that only two of the initial eight general teachers were still part of the study team at that time, while six of the teachers were either on maternity leave or had started teaching a different class. The majority of the new teachers had only received minimal training during a mere one meeting prior to the measurements being conducted in the fall of 2008. Another contributing factor to this drop could be that the extra PE lesson/week introduced in the fall of 2007 was no longer available to the children at the intervention schools. This may have significantly affected the amount of MVPA the children received during school hours. The intervention did not seem to have any effect on physical activity during after-school hours because no significant difference in volume (cpm) or intensity (minutes of MVPA) of physical activity was detected during this time of the day. This seems to be consistent with what Dobbins et al. concluded in their review that there is no evidence for positive effects of school-based physical activity interventions on leisure time physical activity in children [7].

Some of the strengths related to the implementation of this intervention are in line with strengths of comparable intervention studies [14]. First, the results from the teacher interviews during the intervention phase may suggest that empowering the teachers to become effective implementers of positive change in physical activity during school hours may partly explain the increase. Second, the progressive intervention allowed for the possibility of on-site trial and error while the implementers slowly built up their skill set, enabling them to increase school-based physical activity throughout the school day. Third, this method respects a teacher's independence, as it allows individual teachers to adjust the physical activity-related activities at their own will. Finally, it is worth mentioning that the use of accelerometers as an objective measure of school-based physical activity eliminated the possibility of self-report bias, and using hierarchical models to analyze these results is an appropriate technique taking into account the clustered data structure.

There are methodological limitations to this study that must be addressed. Measuring school-based physical activity at the end of each school year, similar to what was done at the beginning of each school year, would likely have provided stronger indications of the intervention progression. Thus, while having three objectively obtained physical activity data points can be viewed as strength, an additional two in the spring of 2007 and spring of 2008 would have strengthened our inferential ability. Despite all the children being a similar age, the study population was relatively small, thus limiting the potential to generalize the findings. Further, we cannot state which specific part of the intervention contributed more than others to the overall increase in physical activity during school hours in the fall of 2007, i.e. if it was the extra PE lesson or integrated physical activity within the various general school subjects. It has also recently been pointed out that having the accelerometers record 60 s epoch is likely to have resulted in a less accurate estimation of physical activity than using shorter epoch like 15 s [39]. This may have underestimated the amount of MVPA the children performed during school hours. Finally, the lack of consensus on where to place accelerometer cut-points defining moderate-to-vigorous physical activity limits our ability to accurately classify physical activity intensity.

## Conclusions

The results suggest that the primary objective of increasing physical activity during school hours was met after one year of intervention, although to a varying degree within the intervention schools and more pronounced among boys. However, no increase in physical activity was observed at the end of the study period, suggesting that any increase in physical activity during school hours may be highly linked to the motivation and training of general teachers. In general boys are more active than girls and may even respond better to school-based intervention compared to girls. Also those with higher BMI are less active during school-hours. Designs of school-based interventions should take this into consideration in order to maximize the effects of increased physical activity among all children.

## Competing interests

The authors declare that they have no competing interests.

## Authors' contributions

KTM carried out the statistical analysis, he contributed to the work involving the study design and he drafted the manuscript. KTM made the greatest contribution to this paper. IS contributed to the study design, project planning, and he was involved in the intervention assessment throughout the study period. TS participated in the statistical analysis and provided guidance during the writing process of this paper. EJ was the project leader and participated in all parts of the work. All authors provided critical revision of the paper and read the final manuscript. All authors read and approved the final manuscript.
